# Effect of consuming a grape seed supplement with abundant phenolic compounds on the oxidative status of healthy human volunteers

**DOI:** 10.1186/s12937-015-0083-3

**Published:** 2015-09-09

**Authors:** Felix Grases, Rafel M. Prieto, Rafel A. Fernández-Cabot, Antonia Costa-Bauzá, Ana M. Sánchez, Marin Prodanov

**Affiliations:** 1 Laboratory of Renal Lithiasis Research, University Institute of Health Sciences Research (IUNICS), University of Balearic Islands, 07122 Palma of Mallorca, Spain; 2 CIBER Fisiopatología de la Obesidad y Nutrición (CB06/03), Instituto de Salud Carlos III, 07122 Palma of Mallorca, Spain; 3 Instituto de Investigación en Ciencias de la Alimentación (CIAL) (CEI, CSIC-UAM), Madrid, Spain

## Abstract

**Background:**

Diverse enzymatic and non-enzymatic antioxidants provide protection against reactive oxygen species in humans and other organisms. The nonenzymatic antioxidants include low molecular mass molecules such as plant-derived phenols.

**Aim of study:**

This study identified the major phenolic compounds of a grape seed extract by HPLC and analyzed the effect of consumption of biscuits enriched with this extract on the urinary oxidative status of healthy subjects by measurement of urine redox potential.

**Methods:**

The major phenolic compounds were characterized in a red grape seed extract separated by HPLC with detection by a photodiode array (PDA), fluorescence (FL) and quadrupole mass spectrometer (MS). A nutritional study in a healthy volunteers group was done. Each volunteer ate eight traditional biscuits with no red grape seed extract supplementation. The second day each volunteer ate eight traditional biscuits supplemented with 0.6 % (wt/wt) of grape seed extract. An overnight urine sample was obtained for each treatment. The redox potential was measured at 25 °C using a potentiometer in each urine sample.

**Results:**

Epicatechin, catechin, procyanidin dimers B1 to B4, and the procyanidin trimer C2 were the major phenolic components in the extract. Epicatechin gallate and procyanidin dimers B1-3-G and B2-3′-G were the major galloylated flavan-3-ols. The forty-six healthy volunteers each shown a reduction of the urine redox potential after the treatment by traditional biscuits supplemented with the grape seed extract.

**Conclusions:**

This simple dietary intervention significantly reduced (33 %) the urine redox potential, reflecting an overall increase in antioxidant status. Incorporation of plant-derived phenols in the diet may increase anti-oxidative status.

## Background

Reactive oxygen species (ROS) and free radicals can react with membrane lipids, nucleic acids, and proteins, leading to cellular damage. Humans and other organisms have diverse antioxidant systems that protect against ROS. Enzymatic antioxidants include superoxide dismutase, glutathione peroxidase, glutathione transferase, and catalase [1]; nonenzymatic antioxidants include low molecular mass molecules, such as ascorbic acid, vitamin E, uric acid, N-acetylcysteine, carotenoids, phenols, and phytates [2]. When ROS are generated in excess or in amounts that overwhelm antioxidant defense mechanisms, oxidative stress and cell damage may occur. Oxidative stress is associated with the pathogenesis of numerous human diseases, including neurodegenerative diseases [3], psychiatric conditions [4], rheumatoid arthritis [5] cancer [6], and renal lithiasis [7]. Thus, there is great emphasis on antioxidant supplement therapy that targets ROS [8].

**Fig. 1:**
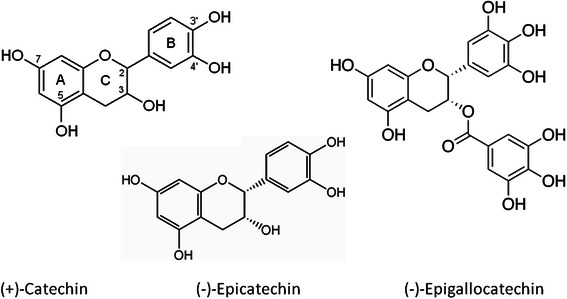
Chemical structure of the elemental flavan-3-ol units of grape seed extracts

Grape (*Vitis vinfera L.*) seed extracts have high levels of numerous anti-oxidants and are considered among the most powerful plant-derived antioxidant foods. Their anti-oxidant activity is mainly attributed to flavonoids and 3 different flavan-3-ols (flavanols): catechin, epicatechin, and epicatechin gallate and its polymers. Flavan-3-ols can donate electrons or protons to ROS and act as scavengers [9, 10]. Some authors [11] claimed that the antioxidant activity of these compounds is due to the presence of hydroxyl groups at positions 3, 5, 4′, and 5′ (and position 7 to a lesser extent) of the benzopyran structure, and those of the gallic acid moiety in the case of the galloylated forms (Fig. 1). Plumb et al. [12] proposed that antioxidant activity increased from monomers to trimers, and then decreased from trimers to tetramers. Grape seed procyanidins are particularly interesting because of the vast diversity of their polymeric and galloylated forms, which are based on several basic elemental units [13].

The main problem in studying individual grape seed procyanidins is their separation and quantification. Normal-phase HPLC can separate flavan-3-ols by molecular mass, but is limited to qualitative or semi-quantitative analysis. Reversed-phase HPLC is mostly used for fine separation of flavan-3-ols, but only allows separation and quantification of monomers, dimers, some nongalloylated trimers, and occasionally tetramers and monogalloylated trimers. In most cases, prior purification of the sample is necessary [14, 15]. Increasing the chromatographic resolution by use of two dimensional chromatography allows separation of heptamer nongalloylated, hexamer monogalloylated, and pentamer digalloylated procyanidins [16], but only provides qualitative results. Even with advances in the development of microbore columns and ultra-high pressure HPLC (UPLC) [17], the main HPLC technique currently used for measurement of flavan-3-ols is based on a C_18_ bonded stationary phase with 5 μm particle size, and this only provides partial quantitative estimates of some individual flavan-3-ols.

Urine antioxidant capacity is usually assessed by complex procedures, including assays of total phenolics (Folin–Ciocalteu assay), ferric-reducing antioxidant power (FRAP), oxygen radical absorbance capacity (ORAC) [18], or by determination of the concentrations of individual antioxidant compounds such as malondialdehyde, ascorbic acid, or uric acid. Measurement of urinary redox potential is a simpler method for assessment of urine antioxidant capacity [19].

In the present study, we determined the major phenolic compounds of exGrape® grape seed extract by an improved HPLC method and analyzed the effect of consumption of this product on urine oxidative status by measurement of urinary redox potential in healthy volunteers.

## Methods

### Extract characterization, reagents, and apparatuses

The reference substances, (+)-catechin (purity > 99 %), (−)-epicatechin (purity > 99 %), (−)-epicatechin-3-gallate (purity > 97.5 %), procyanidin dimer B_1_ (purity > 80 %), and procyanidin dimer B_2_ (purity > 90 %), were from Extrasynthèse (Genay, France). Gallic acid (99 % purity) was from Sigma-Aldrich (St. Louis, Missouri, USA). Procyanidin dimers B_3_-B_7_ were purified from natural extracts by high-speed counter current chromatography (HSCCC) according to Köhler et al. [20] and Esatbeyoglu et al. [21], and were characterized by nuclear magnetic resonance (NMR). Purified procyanidin extract from cocoa (Breko GmbH, Bremen, Germany) was used as a complex reference sample for the identification of procyanidin trimer C_1_ [EC-(4α-8)-EC-(4α-8)-EC], tetramer [EC-(4α-8)-EC-(4α-8)-EC-(4α-8)-EC], pentamer [EC-(4α-8)-EC-(4a-8)-EC-(4α-8)-EC-(4α-8)-EC], and hexamer [EC-(4α-8)-EC-(4α-8)-EC-(4α-8)-EC-(4α-8)-EC-(4α-8)-EC].

Grape seed extract constituents were assessed by the reversed phase (RP) HPLC method of Prodanov et al. [15], with adaptation for a 3 μm particle size stationary phase to increase the separation efficiency [22]. Two HPLC apparatuses were used: *(i)* HPLC/PAD/FL, a Varian 920-LC Galaxie (Varian Instruments, Walnut Creek, California, USA) that was coupled to a photodiode array (PDA) and fluorescence (FL) detectors in series and *(ii)* an Agilent series 1100 HPLC/PAD/MS (Palo Alto, California, USA) that was coupled to a quadrupole mass spectrometer (MS) (Hewlett-Packard series 1100 MSD) with an electrospray interface (ESI). Separation was performed on an ACE-3-C18-AR (200 mm × 4.6 mm, 3 μm particle size) column from Advanced Chromatography Technologies (Aberdeen, UK) and a guard-column (20 × 4.6 mm) of the same material. The mobile phase was a linear gradient of solvent A (water:acetic acid, 98:2) to solvent B (acetonitrile:acetic acid, 98:2) as follows: from 0 to 20 % of B in 80 min, from 20 to 28 % of B in 35 min, from 28 to 100 % of B in 5 min, isocratically 100 % of B for 10 min, from 100 to 0 % of B in 5 min, and equilibration of the column for the next analysis for 10 min. The total run time was 140 min. The flow rate of the mobile phase was 0.6 mL/min. Chromatograms were acquired by measurement of absorption at 280 nm (by the PDA) and fluorescence at 316 nm (273 nm excitation). For ESI, the drying gas was N_2_ (10 L/min at 340 °C), the nebulizing pressure was 40 psi, and the capillary tension was 4000 V. Mass spectra were obtained by scanning negative ions from *m/z* less than 200 at 100 V, *m/z* 200–1000 at 200 V, and *m/z* 1000–2500 at 250 V. Mass spectra were recorded from *m/z* 100 to 2500.

Gallic acid, (+)-catechin, (−)-epicatechin, (−)-epicatechin-3-O-gallate, and procyanidin dimers B_1_ and B_2_ were identified by co-elution and comparison with the masses and UV spectra and retention times of commercial references. Procyanidin dimers B_3_, B_4_, B_5_, and B_7_ were identified by comparison of the UV absorbance spectra, fluorescence following excitation at 273 nm, molecular ions, and retention times of reference substances isolated by HSCCC from natural extracts [20, 21] and characterized by NMR. Procyanidin trimer C_1_ [EC-(4α-8)-EC-(4α-8)-EC], tetramer [EC-(4α-8)-EC-(4α-8)-EC-(4α-8)-EC], pentamer [EC-(4α-8)-EC-(4α-8)-EC-(4α-8)-EC-(4α-8)-EC], and hexamer [EC-(4α-8)-EC-(4α-8)-EC-(4α-8)-EC-(4α-8)-EC-(4α-8)-EC] were identified by comparison of the UV absorbance spectra, fluorescence following excitation at 273 nm, molecular ions, and retention times of the corresponding complex reference sample from a purified procyanidin extract from cocoa [23]. For the procyanidin pentamer [EC-(4α-8)-EC-(4α-8)-EC-(4α-8)-EC-(4α-8)-EC], both the single- and the double-charged ions were present, but for the procyanidin hexamer [EC-(4α-8)-EC-(4α-8)-EC-(4α-8)-EC-(4α-8)-EC-(4α-8)-EC], only the double-charged ion was present in the mass spectra. Procyanidin trimers, C_2_ [C-(4α-8)-C-(4α-8)-C] and [EC-(4α-8)-EC-(4α-8)-C], were identified based on mass, UV absorbance spectra, fluorescence following excitation at 273 nm, retention time, and order of elution [15]. The galloylated procyanidin dimers B_1_-3-G and B_2_-3′-G were identified by mass, UV absorbance spectra, retention times, and order of elution [15].

Gallic acid, (+)-catechin, (−)-epicatechin and (−)-epicatechin-3-gallate were quantified by calibration curves of standards. All procyanidin nongalloylated dimers to hexamers were referred to the calibration curve of the procyanidin dimer B_1_ from the fluorescence chromatograms. The quantities of galloylated procyanidin dimers B_1_-3-G and B_2_-3′-G were calculated based on a calibration curve for the (−)-epicatechin-3-gallate from the 280 nm absorbance chromatograms.

### Human studies

Forty-six non-smokers healthy volunteers (20 males and 26 females, mean age: 34 years, age range: 17 to 60 years) were invited to participate. Individuals who reported consuming antioxidant supplements or omega-3 polyunsaturated fatty acids and those with addiction to alcohol or drugs were excluded. None of the participants received any pharmacological treatment during urine collection. Fruits and vitamin supplements were discontinued 24 h prior to collection of the first urine sample. All subjects were instructed to maintain their usual diets and to not participate in sports activities during the two day study period. The subjects consumed the biscuits with the 100 % of compliance. All subjects provided written informed consent and the study protocol (IB 2030/13 PI) was approved by the Ethics Investigation Committee of the Balearic Islands (Spain).

The first day, each volunteer ate eight traditional biscuits (*Quely* from Quely S.A., Mallorca Islands, Spain) during the day (4 in breakfast and 4 in dinner) with no plant-derived phenol supplementation. The biscuits, of 9 g of weight, are made with wheat, sunflower oil, yeast, olive oil and salt. An overnight urine sample, starting prior to sleep and ending with the first morning urine while still in a fasting state, was obtained the next day.

The second day each volunteer ate eight traditional biscuits supplemented with 0.6 % (wt/wt) of red grape (*Vitis vinfera L.*) seed extract (*Quely Cor* from Quely S.A.,) during the day, corresponding to about 250 mg of phenols, the phenols content of the biscuits was measured by Folin’s method and expressed as gallic acid. This represent a total polyphenol intake about 2 g/d. The red grape seed extract (exGrape®) was purchased from La Gardonnenque-Groupe Grap’Sud® (Cruviers-Lascours, France). The second urine sample was collected on the third morning as described above.

At 2 h after collection of each non filtered urine sample, the redox potential was measured at 25 °C using a Crison potentiometer (Crison Instruments S.A., Barcelona, Spain), with a platinum electrode as the working electrode and a saturated calomel electrode as the reference electrode. Results are shown as means ± standard errors and compared using Student’s paired *t*-test.

## Results

**Fig. 2:**
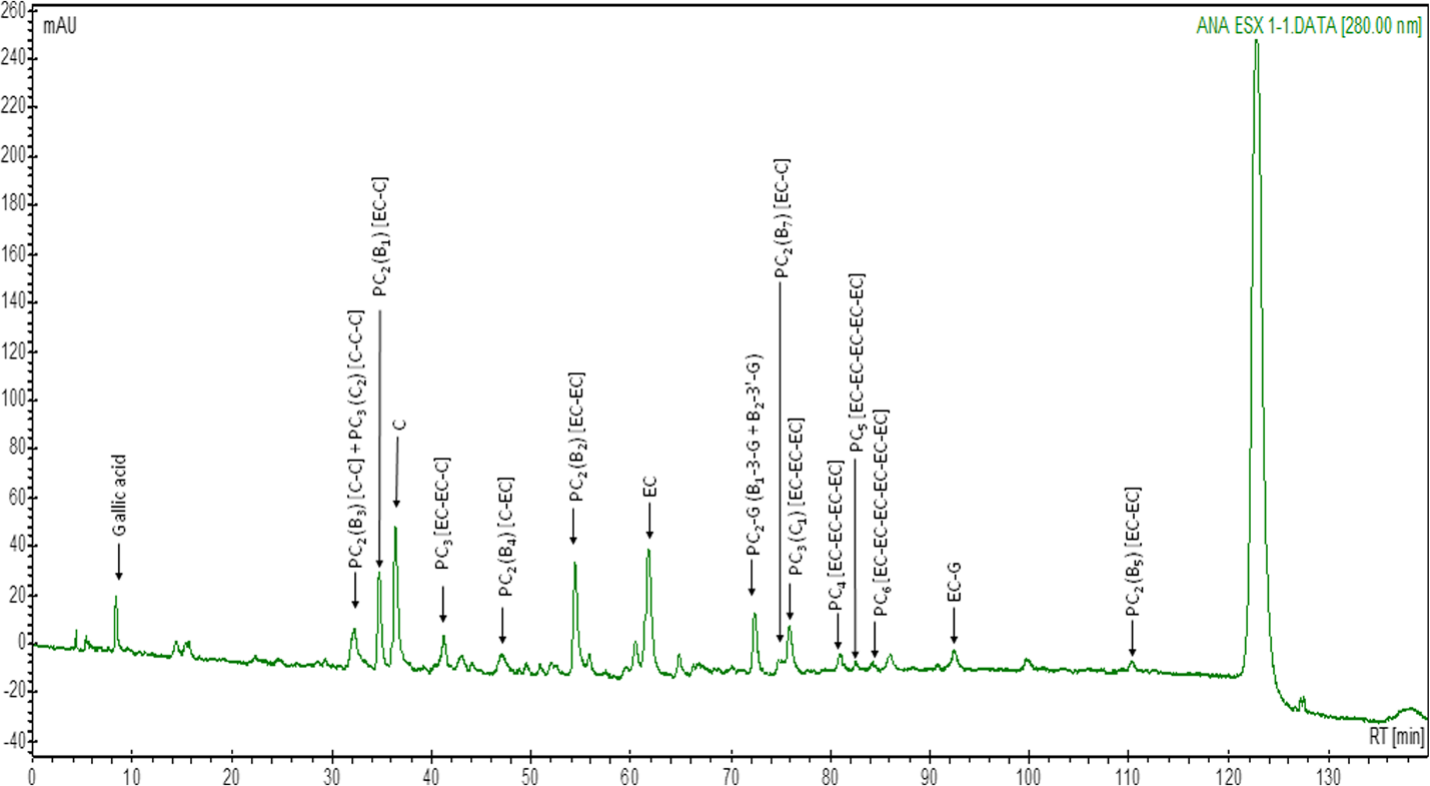
Ultraviolet (280 nm) absorption HPLC chromatogram of the grape seed extract exGrape® (all abbreviations are the same as those shown in Table 1)

Table 1 shows the chemical composition, the mass spectral data and the content of of the grape seed extract exGrape® used in this study. Figure 2 illustrates a typical ultraviolet absorption chromatogram (280 nm) of the studied extract, with indications of the main identified compounds. The results indicates that this extract mainly consists of flavan-3-ol monomers and oligomers (17.46/100 mg), and very small amounts of gallic acid and some other unidentified compounds. Epicatechin, catechin, procyanidin dimers B_1_ to B_4_, and the procyanidin trimer C_2_ were the major components, and these had concentrations of 0.65–1.70/100 g. Lower amounts of other nongalloylated procyanidin trimers and the procyanidin tetramer [EC-EC-EC-EC] were also present. The improved HPLC method detected the procyanidin pentamer [EC-EC-EC-EC-EC] and hexamer [EC-EC-EC-EC-EC-EC], but their amounts were lower than the limit needed for quantification by the fluorescence detector. The improved HPLC method also detected the procyanidin pentamer [EC-EC-EC-EC-EC] and hexamer [EC-EC-EC-EC-EC-EC], but their amounts were also lower than the limit needed for quantification. The total amount of the nongalloylated procyanidin monomers and oligomers was 9.11/100 mg and the amount of the galloylated forms (represented only by the epicatechin gallate and the common peak of the monogalloylated dimers B_1_-3-G and B_2_-3′-G), was 0.65/100 mg.

**Table 1:** Chemical composition, mass spectral data (negative ionisation mode) and content of the main identified constituents of exGrape® seed extract (quantitaive data are reffered to the extrac as it is)

R_t_	Compound	[M-H]^−^	[M-2H]^2−^	Content
(min)		(m/z)	(m/z)	(mg/100 mg)
8.3	Gallic acid	169.0		0.09
32.2	PC_2_ (B_3_) [C-C] + PC_3_ (C_2_) [C-C-C]	577.0, 865.1		0.65
34.8	PC_2_ (B_1_) [EC-C]	577.0		1.19
36.3	C	289.0		1.47
41.3	PC_3_ [EC-EC-C]	865.1		0.30
47.1	PC_2_ (B_4_) [C-EC]	577.1		0.64
54.4	PC_2_ (B_2_) [EC-EC]	577.0		1.69
61.7	EC	289.0		1.70
72.4	PC_2_-G (B_1_-3-G + B_2_-3′-G)	729.1		0.51
74.8	PC_2_ (B_7_) [EC-C]	577.1		0.23
76.0	PC_3_ (C_1_) [EC-EC-EC]	865.1		0.83
81.6	PC_4_ [EC-EC-EC-EC]	1153.1		0.28
83.2	PC_5_ [EC-EC-EC-EC-EC]	1441.1	720.2	0.11^a^
85.0	PC_6_ [EC-EC-EC-EC-EC-EC]	-	864.2	0.02^a^
92.5	EC-G	441.1		0.14
111.2	PC_2_ (B_5_) [EC-EC]	577.2		0.22
	∑ nongalloylated flavan-3-ol monomers			3.17
	∑ nongalloylated flavan-3-ol dimers			4.53
	∑ nongalloylated flavan-3-ol monomers and oligomers			9.11
	∑ galloylated flavan-3-ol monomer and oligomers			0.65
	∑ flavan-3-ols			17.46

*R*_*t*_ retention time, *C* (+)-catechin, *EC* (−)-epicatechin, *PC*_*x*_ procyanidin oligomers (subscript x - number of elemental units (x = 2 (dimer), 3 (trimer), etc.), *PC*_*x*_*G* galloylated procyanidin, *G* galloyl unit

^a^ Values below the limit of quantification

**Fig. 3:**
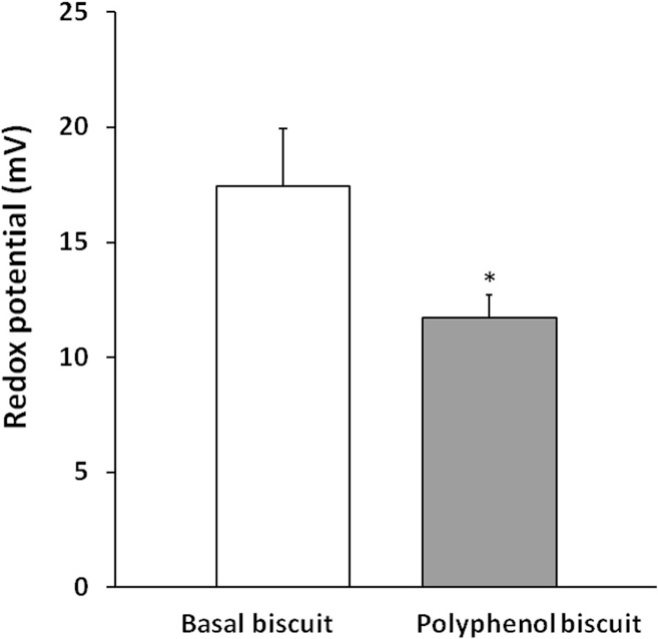
Urine redox potential (mV) of 46 volunteers who ate eight biscuits without phenols on day-1 and eight biscuits supplemented with a red grape seed extract (250 mg polyphenols) on day-2. Values indicate means ± standard errors. * *p* < 0.001 by Student’s paired *t*-test

Figure 3 shows the redox potential of the urine from 46 healthy volunteers at 12 h after consumption of eight traditional biscuits and at 12 h after consumption of eight traditional biscuits with grape seed extract, the redox potential range observed before consuming the phenolic compound rich biscuits was 76 mV and after consuming the phenolic compound rich biscuits of 114 mV. The urinary redox potential was 33 % lower after consumption of the grape seed extract, indicating that consumption of this polyphenol-rich supplement significantly increased the antioxidant capacity of urine.

## Discussion

Table 1 shows that the exGrape® seed extract has a composition typical of grape seed extracts, in that the monomers catechin and epicatechin are most abundant, followed by the nongalloylated procyanidin dimers and trimers with C_4_-C_8_ interflavan bonds [14, 15]. Epicatechin and epicatechin-based oligomers (procyanidin dimer B_2_ and trimer C_1_) are the most abundant among the other catechin-containing derivatives. The natural fluorescence of the nongalloylated flavan-3-ols was greater, so the procyanidin tetramer, [EC-EC-EC-EC], was quantified whereas the other two procyanidin oligomers, [EC-EC-EC-EC-EC] and [EC-EC-EC-EC-EC-EC], were simply separated and detected. To the best of our knowledge this is the first time that pentamer and hexamer procyanidins were separated and detected in a single RP HPLC run with direct injection of an unpurified grape seed extract. The content of flavan-3-ol monomers and oligomers was within the normal limits for this type of extract, even though the level was almost 2-times lower than reported by Shafiee et al. [24] for the same extract 10 years ago. However, the difference with regard to the total content of nongalloylated flavan-3-ol monomers and dimmers was minimal, suggesting that the differences are most probably due to use of different analytical methods. In particular, Shafiee et al. [24] used the vanillin method for assessment of oligomer procyanidins, and this method is based on vanillin condensation of all procyanidins in the sample, including high molecular mass procyanidin polymers. In contrast, we only considered the major and well-resolved procyanidin peaks, and these do not account for all nongalloylated procyanidins.

Grape seed extracts differ from other flavan-3-ol-rich extracts because of their high content of galloylated compounds. In some red varieties of grapes, these can account for up to 18 % of the total procyanidin content [14]. Some authors consider these types of procyanidins as stronger antioxidants than the corresponding nongalloylated analogues [11, 12], and this has led to increased interest in the galloylated forms. Nevertheless, aside from the present study, there is little quantitative data on the levels of galloylated and nongalloylated compounds in grape seed extracts. Thus, even with our improved chromatographic resolution achieved by use of the 3 μm particle size stationary phase, we were only able to separate and unambiguously quantify the epicatechin gallate and a peak containing two monogalloylated dimers, the B_1_-3-G and B_2_-3′-G. Perhaps, the most noteworthy difference we found is that the epicatechin gallate content was 10-fold lower than reported by Shafiee et al. [24] for the same extract. Nevertheless, we should note that we detected many mono- and digalloylated procyanidin oligomers in the extract, but their separation was poor and their amounts were close to or below to limits of detection.

Plant-derived phenols are important dietary antioxidants, and the main dietary sources are fruits and fruit juices. Tea, red wine, vegetables, legumes, cereals, and chocolate also contribute to total polyphenol intake, which may be as high as 1 g/day [25]. Grape seed extract is a concentrated source of polyphenols [26]. Clinical studies found that consumption of polyphenols can contribute to the prevention of cardiovascular disease [27], some types of cancer [28], osteoporosis [29], and calcium oxalate papillary renal stones [30]. The antioxidant activity of polyphenols prevents oxidative membrane damage through metal chelation and scavenging of ROS [24]. The polyphenol content of urine is an indicator of the intake of polyphenol-rich foods, such as cacao [31]. Moreover, total urinary phenols were significantly increased at 4 h after the ingestion of red wine that contained 125 mg of polyphenols [gallic equivalent] [32]. The flavonoid concentration in urine, determined by liquid chromatography–mass spectrometry, may reflect the intake of fruits and vegetables, and the total urinary excretion of flavonoids over 24 h may be a biomarker for fruit and vegetable intake [33].

Urine is the product of blood filtration, and its composition (including that of ions and low molecular mass molecules, such as salts, vitamins, and minerals) mirrors that of blood, despite every subject will have different metabolic rate and therefore different levels of phenolic metabolites could be found in the urine. Thus, a high level of antioxidant tissue destruction may be reflected by decreased urinary excretion of low molecular mass antioxidants. If antioxidants cannot prevent free radical damage or if radical formation is excessive, the oxidant/antioxidant ratio increases and leads to oxidative stress [34]. The urinary total antioxidant capacity determined is dependent on the levels of non-enzymatic antioxidants, which would be predominantly phenolic metabolites in this case, rather than host derived free radicals, oxidants or electrophiles. One method used to determine the antioxidant capacity of a biological fluid, such as urine, is based on the reduction of ferric to ferrous ions; ferrous ions form complexes with specific dyes and generate a product that can be measured spectrophotometrically [35, 36]. The redox potential of an aqueous solution is a measure of its ability to be oxidized or reduced, and a more negative potential indicates a greater reducing capacity. In contrast, strong oxidizing agents have more positive redox potentials, so a decrease in the urine antioxidant status may reflect an increase in urinary redox potential. Individuals with high oxidative stress will therefore excrete urine with low levels of antioxidants and high redox potential. The correlation of the antioxidant capacity of urine, evaluated using ferric ion and the dye 1, 10-phenantroline (FRAP method) [35, 36] with the redox potential using the platinum electrode, was already demonstrated [19]. The correlation was statistically significant by a linear regression equation between antioxidant capacity (mM of Fe^2+^) or FRAP method *versus* the redox potential (mV) (*y* = −0.073*x* + 16.941; R:0.522; p < 0.001) [19]. It demonstrates the suitability of the latter procedure to determine the antioxidant capacity of urine.

The results obtained in this study demonstrate that a single dietary intervention with a grape seed supplement significantly lowered the redox potential of urine, reflecting an overall increase in antioxidant capacity. Other studies have reported similar results after consumption of polyphenol-rich beverages and/or foods [32, 37–39].

## Conclusions

The ExGrape® extract has a composition typical of grape seed extracts, in that the monomers catechin and epicatechin are the most abundant constituents, followed by the nongalloylated procyanidin dimers and trimers with C_4_-C_8_ interflavan bonds. Epicatechin and epicatechin-based oligomers (procyanidin dimer B_2_ and trimer C_1_) are the most abundant among the other catechin-containing derivatives. Among the nongalloylated flavan-3-ols, we quantified the procyanidin tetramer [EC-EC-EC-EC] and separated and detected two other procyanidin oligomers, [EC-EC-EC-EC-EC] and [EC-EC-EC-EC-EC-EC]. The phenolic content of urine is an indicator of the intake of plant-derived phenol-rich foods. A single dietary intervention -- ingestion of biscuits rich in plant-derived phenols -- significantly lowered the redox potential of urine, and this reflects an overall increase in antioxidant capacity. The clinical benefits of more sustained dietary interventions with grape seed extract remain to be established.
